# Postoperative Panophthalmitis Caused by Whipple Disease

**DOI:** 10.3201/eid1505.081209

**Published:** 2009-05

**Authors:** Michel Drancourt, Florence Fenollar, Danièle Denis, Didier Raoult

**Affiliations:** Université de la Méditerranée, Marseille, France; Assistance Publique–Hôpitaux de Marseille, Marseille

**Keywords:** Bacteria, Tropheryma whipplei, Whipple disease, postoperative, uveitis, endophthalmitis, France, letter

**To the Editor:** The clinical spectrum of Whipple disease has widely expanded since its etiologic agent, *Tropheryma whipplei*, was isolated in 2000 (*1*). Systematic 16S rDNA sequencing unexpectedly identified *T*. *whipplei* in patients for whom blood cultures were negative for endocarditis, spondylitis, and uveitis (*2*). Features common to these conditions and to Whipple disease include long-standing, unexplained arthralgia and deterioration of the patient’s condition after treatment with immunosuppressive drugs (*2*). We report an unexpected case of postoperative panendophthalmitis identified by systematic 16S rDNA sequencing of a vitreous sample in a patient who had unexplained arthralgia and had been given topical corticosteroids after cataract surgery.

A 78-year-old woman in France underwent left eye phacoemulsification with intraocular lens implantation in May 2005 and retinal surgery followed by local corticoid application in April 2006. She had experienced cortisone-resistant polyarthralgia for 2 years before the first surgery. In July 2006, she showed decreased visual acuity (20/1,000) and a painful, red eye. Chronic postoperative endophthalmitis was suspected, and the patient underwent anterior chamber paracentesis (ACP). Parameters included 0.614 × 10^9^ eosinophils/L in the blood and an erythrocyte sedimentation rate of 70 mm in the first hour.

Sequencing of 16S rDNA of the ACP specimen showed 99.9% similarity with that of *T*. *whipplei* (GenBank accession no. AJ551273). A specific PCR confirmed this result in the ocular sample and detected *T*. *whipplei* in saliva and stool samples, whereas blood and cerebrospinal fluid were negative for the organism by PCR. Duodenal biopsy specimens were negative by periodic acid–Schiff staining, specific immunohistochemical analysis, and PCR.

The patient was treated with topical corticosteroids, cycloplegic drugs, doxycycline (200 mg/d), hydroxychloroquine (200 mg 3×/d), and sulfamethoxazole/trimethoprim (1,600 mg and 320 mg 3×/d) (*2*). She was hospitalized for 7 days in the ophthalmology department and for 4 days in the infectious disease department. At 8-month follow-up, visual acuity had improved (20/50) despite intraocular inflammation with a Tyndall effect, moderate capsular opacification, decreased vitreitis, macular edema, and retinal macular abnormalities shown by optical coherence tomography. *T*. *whipplei* DNA was again not detected by PCR in saliva and stool samples at 8-month follow-up, and the patient remained free of symptoms at 16-month follow-up when treatment was stopped.

Diagnosis of Whipple disease uveitis was confirmed by detection of *T*. *whipplei* DNA in the ocular sample by 2 laboratories that used 2 molecular targets and negative controls. *T*. *whipplei* was identified by 16S rDNA sequencing and by detection of *T*. *whipplei*–specific repeat sequences. Further investigations detected *T*. *whipplei* in saliva and stool samples. Uveitis was the initial manifestation of Whipple disease, although patient evaluation showed a 2-year history of idiopathic, corticoresistant polyarthralgia described as a hallmark of Whipple disease (*2*). Initial unexplained eosinophilia in blood was observed, as in several confirmed cases of Whipple disease (*2*).

Uveitis has been reported in Whipple disease (*2*), but <20 patients had *T*. *whipplei* in a diseased eye (Table). *T*. *whipplei* has been found by periodic acid–Schiff staining of foamy macrophages, electron microscopy, and immunocytochemical detection in ocular monocytes (*3*–*10*). Detection of *T*. *whipplei* DNA (*6*–*10*) has been confirmed by sequencing in only 2 patients, including the case reported herein.

**Table:** Characteristics of 19 patients with Whipple disease uveitis documented by presence of *Tropheryma whipplei* in a diseased eye*

Patient. no.	Age, y/sex	Class	Location	Postoperative uveitis	Use of local or systemic steroids	Microscopy, PAS stain	EM	PCR	Reference
1	52/M	I	B	–	No	+	+	ND	(*3*)
2	60/M	A	B	–	No	+	+	ND	(*5*)
3	56/M	A	B	+	Yes	+	ND	ND	(*4*)
4	47/M	A	B	–	Yes	+	ND	ND	(*4*)
5	65/M	A	B	+	Yes	+	+	+	(*6*)
6	59/F	A	B	+	Yes	+	+	+	(*7*)†
7	53/F	Pa	U	–	Yes	+	ND	+	(*7*)†
8	65/M	I	U	+	Yes	+	ND	+	(*7*)†
9	NR/NR	NR	NR	NR	NR	ND	ND	+	(*8*)
10	65/M	I	U	+	Yes	ND	ND	+‡	(*7*)
11	81/M	P	U	+	Yes	ND	ND	+‡	(*7*)
12	35/M	P	U	NR	NR	ND	ND	+‡	(*7*)
13	46/M	P	U	–	No	ND	ND	+‡	(*7*)
14	3/F	A	U	–	No	ND	ND	+‡	(*7*)
15	90/F	A	U	+	Yes	ND	ND	+‡	(*7*)
16	69/M	P	U	+	Yes	ND	ND	+‡	(*7*)
17	20/F	A	U	+	Yes	ND	ND	+‡	(*7*)
18	74/F	Pa	U	+	Yes	ND	ND	+‡	(*7*)
19	78/F	P	U	+	Yes	ND	ND	+‡	(*7*)

*PAS, periodic acid–Schiff; EM, electron microscopy; I, intermediate; B, bilateral; ND, not done; A, anterior; Pa, panuveitis; U, unilateral; NR, not reported; P, posterior. †Reviewed by Drancourt et al. (*7*). ‡These patients were considered to have suspected cases.

Diagnosis of *T*. *whipplei* uveitis in our patient was made 3 months after ocular surgery. The patient’s condition was diagnosed as chronic postoperative panendophthalmitis, which raised the issue of nosocomial transmission of *T*. *whipplei*. We have reported a correlation between diagnosis of *T*. *whipplei* uveitis and a history of ocular surgery (*7*). By reanalyzing detailed published reports, we found that 11 of 19 patients with intraocular demonstration of *T*. *whipplei* had a history of ocular surgery before documentation of Whipple disease uveitis (Table). *T*. *whipplei* has not been reported as being responsible for nosocomial infection. Items used during the patient’s ocular surgery were confirmed to be disposable and nonreused.

Topical drops of corticosteroids commonly applied during cataract surgery for intraocular lens implantation penetrate ocular structures. An alternative hypothesis is that corticosteroids applied during ocular surgery reactivate a latent ocular infection. Our review indicated that 13 of 19 patients with documented *T*. *whipplei* uveitis had received topical or systemic corticosteroids before the diagnosis (Table) (*7*). Worsening of Whipple disease has been reported in patients receiving corticoid therapy for arthralgia (*10*). We speculate that our patient had an asymptomatic ocular infection before surgery.

This case shows that ocular surgery and use of topical corticosteroids that penetrate ocular structures could reactivate a latent *T*. *whipplei* ocular infection. We suggest that patients with postoperative panendophthalmitis be tested for *T*. *whipplei* by PCR.
